# Is Mental Health Worse in Medical Students than in the General Population? A Cross-Sectional Study

**DOI:** 10.3390/medicina60060863

**Published:** 2024-05-25

**Authors:** Giuseppe Stirparo, Roberta Pireddu, Marta D’Angelo, Dario Bottignole, Riccardo Mazzoli, Luca Gambolò

**Affiliations:** 1 SIMED (Società Italiana di Medicina e Divulgazione Scientifica), 43125 Parma, Italy; 2 School of Public Health, Vita-Salute San Raffaele University, 20132 Milano, Italy

**Keywords:** students, anxiety, depression, suicide, quality of life

## Abstract

*Background and Objectives*: In recent years, there has been a notable increase in university students experiencing severe mental illness. The transition to university life can be demanding, leading to mental health disorders. Persistent stress and anxiety can cause demotivation, difficulties with concentration, cognitive impairment, and reduced academic performance. Mental health issues can also impact social relationships and overall well-being. This cross-sectional study aims to investigate the mental health of medical students and compare it with the mental health of the non-student population. *Materials and Methods:* The survey collected demographic information such as age and gender. Participants were questioned about their self-perceived mental distress, diagnosed mental disorders, and history of therapy for mental distress. Various validated assessment tools were utilized to assess mental health and quality of life. *Results*: Medical students exhibit a higher self-perception of mental symptoms that does not translate into a significantly higher prevalence of diagnosed mental disorders. Medical students experience higher levels of anxiety and subclinical depressive symptoms and lower quality of life. Female participants reported lower QoL scores and higher levels of anxiety symptoms compared with male participants. While the prevalence of mental disorders did not differ significantly between genders (except for clinical anxiety), females tended to perceive a higher burden of mental health challenges. *Conclusions:* By addressing mental health issues among medical students, especially females, institutions can create a more supportive and conducive learning environment. Encouraging open conversations about mental health and providing accessible mental health services can help in destigmatizing mental health challenges and promoting early intervention when needed.

## 1. Introduction

The period from adolescence to adulthood is a time of rapid growth and change, presenting various challenges for students. Academic pressure, social adjustments, financial burdens, relationships, and adapting to new environments can have a significant impact on their well-being and contribute to mental health issues [1,2,3,4]. The World Health Organization (WHO) states that mental disorders often emerge before the age of 24, emphasizing the critical nature of college years for mental health development [5,6]. Despite the perception of college students as privileged [7], mental health problems affect students globally, regardless of their socioeconomic status [8,9,10,11,12,13]. University students experiencing severe mental illness is a growing public health problem [14]. Research shows that the transition to university life can be demanding, leading to heightened stress, anxiety, depression, and other mental health disorders [6]. Persistent stress and anxiety can result in burnout, causing demotivation, difficulties with concentration, cognitive impairment, and reduced academic performance [15,16,17]. Mental health issues can also impact social relationships and overall well-being. Tragically, mental health problems can even contribute to suicide, which is one of the leading causes of death among college students [18,19].

The college years coincide with a critical developmental period toward adulthood. Mental disorders during this time can have long-term consequences, affecting an individual’s physical and psychological health, leading to chronic problems [20], and impairing relationships [21]. Therefore, it is crucial to prioritize mental health and wellness initiatives in universities to support academically successful and emotionally resilient students.

A meta-analysis of 195 studies involving 129,123 medical students in 47 countries found that 27.2% screened positive for depression and 11.1% reported suicidal ideation during medical school. Shockingly, only 15.7% of those who screened positive sought treatment [22]. 

In student populations, depression symptoms were associated with factors such as female gender, academic stress, smoking, traumatic experiences, alcohol use, poor sleep quality, financial strain, and low self-confidence [1]. Similarly, anxiety symptoms in students were linked to being female, poor sleep quality, irregular eating habits, internet addiction, alcohol consumption, low self-esteem/self-efficacy, and academic difficulties [1].

We aim to investigate the mental health of medical students and compare it with the mental health of the non-student population. Despite the growing recognition of mental health issues among university students, there remains a lack of research that directly confronts the differences in mental health between this population and individuals who are not enrolled in higher education. By addressing this research gap, we seek to understand the unique mental health challenges faced by university students and explore potential implications for their overall well-being and academic performance. The findings of this study can contribute to the development of targeted mental health interventions and support programs to promote the emotional resilience and success of college students during this critical developmental phase.

## 2. Materials and Methods

This case–control study was approved by the SIMED research council and followed the principles outlined in the Declaration of Helsinki. To protect participants’ privacy, all data were anonymized. 

After careful consideration, we selected three cases for every control due to the ease of accessibility and calculated the required sample sizes for each group. Utilizing the R statistical software (version 4.3.3), we set a power of 85% for detecting a medium effect size (d = 0.500), maintaining a sample size ratio of 3:1 between cases and controls. This approach was chosen to enhance the robustness of our findings while efficiently utilizing the available resources. 

The survey was developed using Google Forms and distributed via SIMED’s social network pages between May and June 2023 to recruit 50 workers and 150 medical students from various Italian universities. Once the desired number of participants was reached, the survey was closed. 

The survey questionnaire collected demographic information, including age and gender. Participants were asked if they thought they had a mental disorder and if they had a diagnosed mental disorder. Additionally, they were inquired about whether they had ever undergone therapy for mental distress.

Several validated assessment tools were employed to evaluate mental health and quality of life. The questionnaires were meticulously selected from those validated in Italian, with a robust utilization documented in the literature and a preference for validated cutoff scores. The choice was made through comprehensive discussions among the authors, carefully considering a balance between the depth of assessment and the number of items to ensure the methodological integrity and relevance of the instruments employed.

The Irritability, Depression, and Anxiety Scale (IDAS) was used to assess participants’ levels of irritability, depression, and anxiety [23]. The Scale for Suicide Ideation (SSI) was utilized to gauge propensity toward suicidal thoughts [24]. For measuring quality of life (QoL), the well-being index, known as the World Health Organization—Five Well-Being Index (WHO-5), was employed [20]. Clinical cutoff points from the existing literature were adopted to categorize participants. Scores equal to or above 8 on the IDAS were considered clinically significant for irritability, scores equal to or above 7 were considered clinically significant for depression, and scores equal to or above 9 were considered clinically significant for anxiety [23]. A cutoff point of 13 on the WHO-5 indicated a sufficient quality of life [25], while a score of 4 or higher on the SSI suggests a risk of suicide [26].

Statistical analysis was conducted using the R programming language [27]. Categorical values were summarized as frequencies and percentages, while continuous variables were presented as the mean and standard deviation. To compare categorical values between workers and medical students, the chi-square test was employed. Additionally, the t-test was used to compare continuous variables between the two groups. We employed linear regression analysis to examine the impact of various sociodemographic and psychopathological variables on quality of life.

This study adhered to the Strengthening the Reporting of Observational Studies in Epidemiology (STROBE) statement to ensure the comprehensive and transparent reporting of its findings [28].

## 3. Results

After distributing the survey through SIMED’s social network pages, we successfully recruited 150 medical students from the cities of Reggio Emilia, Parma, and Milan and 50 workers for participation from Parma, Milan, and Piacenza. After data collection, we encountered missing values in the responses. As per standard data cleaning procedures, we removed incomplete entries, resulting in a final sample size of 124 medical students and 43 workers for the statistical analysis. Based on the results of the Levene test, it was determined that the test was non-significant for all the variables. 

Table 1 displays sociodemographic and anamnestic information of participants.

Of the 38 students in therapy, 17 are taking medication, and 21 are undergoing psychotherapy.

**Figure 1:**
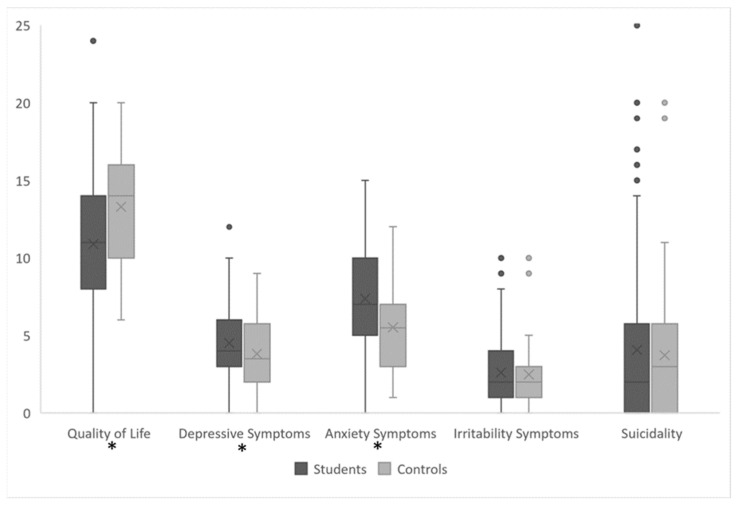
Differences in psychopathology among students and workers. Significant differences are marked (*).

For the variable “clinical irritability,” we encountered a situation where the frequency in a contingency table was less than five, so we used Fisher’s exact test instead of the regular chi-square test. Quality of life demonstrated a statistically significant discrepancy, with an overall mean QoL score of 11.60 (SD = 4.28), compared to 11.00 (SD = 4.23) for medical students and 13.30 (SD = 3.98) for workers (*p* = 0.002, d[CI95%] = 0.556 [0.187; 0.920]). A similar pattern was observed in the prevalence of poor QoL, where a higher percentage of workers (41.9%) reported poor QoL, in contrast to 66.1% of medical students (*p* = 0.005, OR[CI95%] = 2.71 [1.33; 5.52]). The mean depression score for the total sample was 4.52 (SD = 2.36), with medical students scoring 4.77 (2.30) and workers scoring 3.79 (2.40) (*p* = 0.018, d[CI95%] = −0.423 [−0.779; −0.062]). However, clinical depression rates were not significantly different between medical students (23.4%) and workers (18.6%) (*p* = 0.515, OR = 1.34 [0.558; 3.200]). Anxiety scores demonstrated a significant variance, with medical students scoring 7.70 (SD = 3.24), workers scoring 5.51 (SD = 2.85), and an overall mean of 7.14 (SD = 3.28) (*p* < 0.001, d[CI95%] = −0.697 [−1.071; −0.316]). Clinical anxiety showed a distinct contrast, as 34.7% of medical students and only 11.6% of workers were affected (*p* = 0.004, OR = 4.03 [1.480; 11.000]). Irritability scores exhibited an overall mean of 2.66 (SD = 2.40), with medical students scoring 2.74 (SD = 2.44) and workers scoring 2.44 (SD = 2.27) (*p* = 0.481, d[CI95%] = −0.125 [−0.472; 0.224]). Similarly, the clinical irritability rates remained consistent between medical students (6.5%) and workers (4.7%) (*p* = 1.000 Fisher, OR = 1.41 [0.288; 6.930]). The mean suicidality score was 3.96 (SD = 4.98), with medical students scoring 4.06 (SD = 5.09) and workers scoring 3.67 (SD = 4.69) (*p* = 0.666, d[CI95%] = −0.077 [−0.423; 0.271]). Moreover, being at risk of suicide was comparable, affecting 39.5% of medical students and 37.2% of workers (*p* = 0.789, OR = 1.10 [0.539; 2.250]). Figure 1 displays differences in psychopathology between students and workers.

We employed linear regression analysis (refer to Table 2) to investigate the predictive relationship between various factors and quality of life. The key predictors examined were sex, age, depression, anxiety, irritability, and suicidality. Our findings reveal significant insights into the impact of these variables on students’ QoL.

We analyzed biological sex differences in psychopathology among students, categorizing them into male and female groups. To comprehensively understand the variations in psychopathological characteristics between the two genders, we used both a dimensional and categorical perspective. For the variable “clinical irritability,” we encountered a situation where the frequency in a contingency table was less than five, so we used Fisher’s exact test instead of the regular chi-square test.

A comparison between male (N = 42) and female (N = 82) participants revealed no significant age difference (*p* = 0.189, d[CI95%] = −0.252 [−0.625; 0.123]). While a higher percentage of females (53.7%) believed they had mental disorders compared to males (40.5%), the difference was not statistically significant (*p* = 0.165, OR [CI95%] = 1.70 [0.801; 3.620]). Similarly, the prevalence of diagnosed mental disorders showed no significant gender difference, with 20.7% of females and 11.9% of males (*p* = 0.223, OR [CI95%] = 1.94 [0.660; 5.670]). Rates of receiving mental health treatment were also similar, with 32.9% of females and 26.2% of males reporting treatment (*p* = 0.441, OR [CI95%] = 1.38 [0.605; 3.170]). Among the 21 treated males in this study, 8 individuals were receiving treatment through psychiatric drugs, while 17 were engaged in psychotherapy. On the other hand, out of the 27 treated females, 13 were undergoing psychiatric drug treatment, and 14 were involved in psychotherapy sessions.

**Figure 2:**
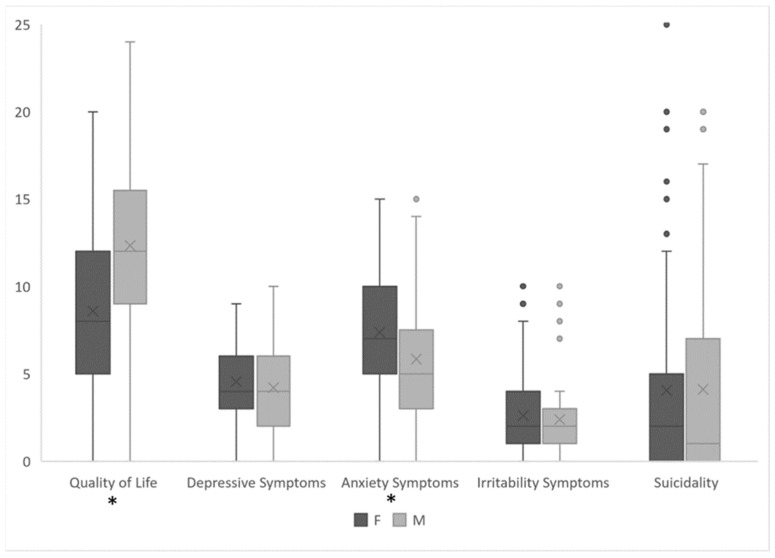
Gender differences in psychopathology among students. Significant differences are marked (*).

Females exhibited significantly lower quality of life scores (M = 10.06, SD = 3.90) compared to males (M = 12.86, SD = 4.26) (*p* < 0.001, d[CI95%] = 0.694 [−1.079; −0.305]), and a greater proportion of females (73.2%) reported poor quality of life compared to males (52.4%) (*p* = 0.021, OR [CI95%] = 2.48 [1.14; 5.40]). The depression scores were not significantly different between genders (*p* = 0.150, d[CI95%] = 0.275 [−0.100; 0.649]); the clinical depression rates were not statistically different between males (19.1%) and females (25.6%) (*p* = 0.414, OR [CI95%] = 1.46 [0.585; 3.66]). Females had higher anxiety scores (M = 8.62, SD = 2.57) than males (M = 5.90, SD = 3.66) (*p* < 0.001, d[CI95%] = −0.860 [0.512; 1.307]), with clinical anxiety affecting 42.7% of females and 19.1% of males (*p* = 0.009, OR [CI95%] = 3.16 [1.31; 7.67]). Irritability scores showed no significant gender difference (*p* = 0.308, d[CI95%] = 0.194 [−0.180; 0.567]), and the clinical irritability rates were similar (6.1% for females, 7.1% for males; *p* = 0.823; OR [CI95%] = 1.000 [0.192; 3.720]). Suicide scores and risk of suicide were comparable between genders (*p* = 0.969, d[CI95%] = −0.040 [−0.412; 0.332] for scores; *p* = 0.314¸ for risk, OR [CI95%] = 1.49 [0.685; 3.240]). Figure 2 illustrates the disparities in psychopathology observed between male and female students.

## 4. Discussion

This study provides valuable and insightful observations regarding the psychopathological and well-being measures among medical students and workers. 

The results demonstrated that a higher percentage of medical students (49.2%) perceived themselves as having mental disorders, in contrast to 27.9% of the control group. Medical students’ exposure to mental health-related knowledge and resources could contribute to their heightened recognition of potential psychological concerns; on the other hand, the higher self-perception of mental disorders among medical students may also be influenced by the presence of subclinical symptoms. The challenging and competitive environment of medical education may trigger mild depressive symptoms, heightened anxiety, or elevated stress levels, contributing to the perception of mental health concerns.

While the study revealed a disparity in self-perception, there were no statistically significant differences in the prevalence of diagnosed mental disorders between medical students (17.7%) and workers (7.0%). This finding suggests that despite medical students’ heightened awareness of their mental well-being, the formal diagnosis of mental disorders did not significantly differ between the two groups. 

Particularly noteworthy is the finding that medical students exhibited significantly worse quality of life (QoL) compared to their counterparts. Moreover, the data also revealed significantly higher levels of anxiety symptoms among medical students, indicating an elevated risk of experiencing subclinical and clinically significant anxiety compared with the other group. 

While the study did not find statistically significant differences in the prevalence of clinical depression and suicidality between medical students and workers, medical students reported higher levels of depressive symptoms. Subclinical depressive symptoms and mental distress can exert a significant impact on an individual’s quality of life and functioning, even without reaching the threshold for a formal clinical diagnosis. 

The results obtained from the linear regression analysis provide valuable insights into the predictors of quality of life (QoL) among the students. Biological sex emerged as a meaningful predictor, with female participants reporting lower QoL scores compared with their male counterparts. Moreover, the results revealed that both depressive symptoms and anxiety had substantial negative impacts on QoL. These findings are consistent with previous research, emphasizing the harmful effects of depression and anxiety on various aspects of life satisfaction and psychological functioning [29].

We found a significant difference in QoL between males and females, with female participants reporting lower QoL scores. Regarding mental health perceptions, a higher percentage of female participants believed they had mental disorders compared with males, although the difference was not statistically significant. However, the study did not find a significant difference in the prevalence of diagnosed mental disorders between males and females. This may suggest that while females perceive a higher burden of mental health challenges, formal diagnoses are relatively similar between genders. This raises questions about the factors influencing the perception of mental health issues and the subsequent decision to seek professional diagnosis and treatment. While there was no significant difference in depression scores between males and females, female participants showed higher levels of anxiety symptoms compared with their male counterparts. This finding aligns with previous research indicating higher prevalence rates of anxiety disorders among females [30,31].

The higher prevalence of clinical anxiety among females further corroborates these findings, suggesting that females may be more susceptible to experiencing clinically significant anxiety symptoms. 

It is very important to analyze the differences between the mental health of students and the general population because it may implement support protocols for medical students or mental health promotion in universities. 

It is very complex to understand whether there is a selection of the university population at the base or whether anxiety is generated due to the university environment. Despite this, we still need to continue to analyze this phenomenon with further studies, and we need to optimally understand strategies to reduce this phenomenon. Mental well-being is an important element in ensuring that students have a healthy path to the end of their university studies. 

In addition, a very important element is that our analysis does not consider COVID-19, which is a very important factor in the development of disease in different social groups [32,33]; it may also have influenced it differently depending on occupation. Some groups may have been more affected than others in terms of social discomfort. 

We should point out that the results of this type of study are very challenging due to the difficulty of recruiting participants. In fact, as is well known with regard to studies developed using questionnaires, it is difficult to involve a representative sample of the population, and they are generally not interested in participating [34]. In this population, these subjects may present clinically relevant characteristics and pathological profiles for study.

Despite this, in our study, we recorded such a significant difference that it was enough to collect a number of questionnaires with a 3:1 ratio of cases to workers. This element is very important and allowed us to record these large differences with even a small sample and with an apparent imbalance between case and control. But the large difference recorded made it unnecessary to pair the two samples, also in light of the minor sociodemographic differences, as presented in Table 1. The demographic similarities and the large difference between the two groups emphasize that the differences observed, despite the reported limitations of our study, are nonetheless important and similar to reality.

It is essential to acknowledge some limitations that may impact the interpretation of our results:

1. Bias in Participant Selection: Voluntary participation may attract individuals who are more inclined to discuss their mental health. However, this bias should be balanced between the two groups.
2. Medium Sample Size: The sample size of the study was moderate, which may limit the generalizability of the findings to a broader population. A larger and more diverse sample would provide more robust and representative results.
3. Cross-Sectional Design: This design does not allow for establishing causal relationships or the assessment of changes in mental health over time. Longitudinal studies are necessary to accurately understand the trajectories of mental health in medical students and workers.
4. Reliance on Short Scales: While these scales provide a quick assessment, they may lack the depth and comprehensive evaluation of more extensive instruments. Additionally, relying on short scales may not adequately capture all the dimensions of mental health.
5. Lack of In-Depth Assessment of Social Support or Academic Year: The study does not thoroughly explore the role of social support in influencing mental health outcomes. Social support is a critical factor in an individual’s well-being, and its absence or presence can significantly impact mental health. In the future, it would be interesting to investigate the effect of both factors.

One of the notable strengths of our study lies in its direct comparison between medical students and the general population, a comparative approach that is not frequently explored in the literature on medical student well-being. Moreover, our examination of subclinical symptoms, often overlooked in psychopathological studies, adds a novel dimension to our investigation. By addressing these aspects, we contribute valuable insights into the nuanced experiences of medical students and shed light on potentially underrepresented areas of psychopathology. Additionally, our predictive model demonstrates substantial explanatory power, elucidating approximately 50% of the variability in QoL. 

However, we should emphasize that the study we have developed has strong territorial influences. Indeed, many elements such as years of university study can influence the mental health of students. This is why it might be desirable to disseminate studies such as these in various European countries because each country has different organizational models, including university support, both economic and psychological, which can have a marked influence on students’ health. Sharing the best models for reducing this phenomenon may be important [35].

## 5. Conclusions

The present study sheds light on the psychopathological challenges medical students face, and the importance of recognizing and addressing subclinical mental health symptoms. Medical students exhibit a higher self-perception of mental disorders. This heightened awareness, however, does not translate into a significantly higher prevalence of diagnosed mental disorders, indicating a discrepancy between self-perception and formal diagnoses. The higher perception of mental health problems among medical students may be attributed, at least in part, to the presence of subclinical symptoms; they may not be severe enough to be classified as a diagnosable disorder, but they can still significantly impact an individual’s well-being and functioning.

One of the most noteworthy findings is the lower quality of life (QoL) reported by medical students, especially compared to their counterparts. The demanding and competitive nature of medical education (and in more general terms of university) may contribute to increased stress and reduced well-being among students.

Female students reported lower QoL scores and higher levels of anxiety symptoms compared with male participants. While the prevalence of diagnosed mental disorders did not differ significantly between genders (except for clinical anxiety), females tended to perceive a higher burden of mental health challenges. The higher levels of anxiety and depressive symptoms in medical students and their effect on QOL underscore the need for proactive mental health support in this population, particularly among female medical students, to ensure early identification and intervention.

Recognizing subclinical mental health symptoms is crucial because they can have a significant impact on individuals’ quality of life and overall psychological well-being, even without reaching the threshold for a formal clinical diagnosis. Mental distress can affect academic performance, personal relationships, and career prospects. Therefore, universities and medical institutions should prioritize mental health support and resources, including counseling services and educational programs to reduce stigma and promote well-being.

By proactively addressing mental health issues among medical students, especially among females who may be more susceptible to experiencing clinically significant anxiety symptoms, institutions can create a more supportive and conducive learning environment. In addition, encouraging open conversations about mental health and providing accessible mental health services can help in destigmatizing mental health challenges and promoting early intervention when needed.

## Figures and Tables

**Table 1:** Sociodemographic and anamnestic information of participants.

	Total (N = 167)	Medical Students (N = 124)	Workers (N = 43)	*p*	ES[CI95%]
Age (y) M(SD)	25.1 (5.1)	24.2 (4.3)	27.5 (6.2)	<0.001	D = 0.671 [0.293; 1.040]
Male N(%)	62 (37.1)	42 (33.9)	20 (46.5)	0.139	OR = 0.589 [0.291; 1.190]
Do you think you have mental disorders? (y) N(%)	73 (43.7)	61 (49.2)	12 (27.9)	0.015	OR = 2.50 [1.18; 5.31]
Do you have diagnosed mental disorders? (y) N(%)	25 (15.0)	22 (17.7)	3 (7.0)	0.135	OR = 2.88 [0.815; 10.100]
Are you being treated for mental problems? (y) N(%)	51 (30.5)	38 (30.6)	13 (30.2)	0.960	OR = 1.02 [0.479; 217]

**Table 2:** Linear regression results for predicting quality of life (QoL).

**Linear Regression to Predict QoL**				
Adaptation of Models				
Model	R^2^		*p*	
1 (Age)	0.016		0.159	
2 (Age; Biological Sex)	0.126		<0.001	
3 (Age; Biological Sex; Psychopathology)	0.502		<0.001	
**Anova**				
	Sum of Squares	df	F	*p*
Age	3.39	1	0.362	0.549
Biological Sex	39.51	1	4.221	0.042
Depression	236.29	1	25.246	<0.001
Anxiety	82.22	1	8.784	0.004
Irritability	4.25	1	0.454	0.502
Suicidality	2.46	1	0.263	0.609
**Model Coefficients**				
Predictor	Estimate	SE	T	*p*
Intercept	18.906	1.656	11.415	<0 .001
Age	−0.040	0.066	−0.602	0.549
Biological Sex: F-M	−1.359	0.662	−2.054	0.042
Depression	−0.755	0.150	−5.024	<0 .001
Anxiety	−0.357	0.121	−2.964	0.004
Irritability	−0.085	0.126	−0.674	0.502
Suicidality	−0.033	0.065	−0.513	0.609

## Data Availability

The data presented in this study are available on request from the corresponding author. The data are not publicly available in accordance with national data safety guidelines.
